# Primary Lymphocutaneous *Nocardia brasiliensis* in an Immunocompetent Host: Case Report and Literature Review

**DOI:** 10.3390/medicina58040488

**Published:** 2022-03-28

**Authors:** Igor Dumic, Alethea Brown, Kyle Magee, Sammer Elwasila, Marija Kaljevic, Marina Antic, Oladapo Igandan, Milena Cardozo, Libardo Rueda Prada, Margaret Paulson

**Affiliations:** 1 Hospital Medicine, Mayo Clinic Health System, Eau Claire, WI 54703, USA; igandan.oladapo@mayo.edu (O.I.); cardozo.milena@mayo.edu (M.C.); prada.libardo@mayo.edu (L.R.P.); paulson.margaret@mayo.edu (M.P.); 2 Mayo Clinic College of Medicine, 200 1st St SW, Rochester, MN 55905, USA; 3 Hospital Medicine, Mayo Clinic, Jacksonville, FL 32224, USA; brown.alethea@mayo.edu; 4 Department of Microbiology, Mayo Clinic, Jacksonville, FL 32224, USA; magee.kyle@mayo.edu; 5 Department of Infectious Disease, Mayo Clinic, Jacksonville, FL 32224, USA; elwasila.sammer@mayo.edu; 6 Department of Hospital Medicine, University of Connecticut, Hartford, CT 06032, USA; m.kaljevic@yahoo.com; 7 Icahn School of Medicine at Mount Sinai, 1 Gustave L. Levy Pl, New York, NY 10029, USA; marina.antic@outlook.com

**Keywords:** *Nocardia brasiliensis*, lymphocutaneous, cellulitis

## Abstract

*Nocardia* spp. is a Gram-positive, partially acid-fast aerobic bacterium usually associated with infection in immunocompromised people. The most common sites of infection are the skin, lungs, and the brain, however disease can disseminate and affect every organ. Clinical manifestations of cutaneous disease are varied and frequently misdiagnosed. We present a case of an immunocompetent 66-year-old man who sustained a left finger injury while gardening. He was misdiagnosed on several occasions and treated with inappropriate antibiotics against *Streptococcus* spp. and *Staphylococcus* spp. When infection spread cutaneously, sporotrichoid (lymphocutaneous) nocardiosis was suspected and the patient was started on appropriate therapy with Bactrim which resulted in a cure. We also summarize the literature on lymphocutaneous infection by *Nocardia brasiliensis*. By reporting this case, we want to raise awareness among clinicians about unusual causes of cellulitis, the differential diagnosis of lymphocutaneous infection and the importance of obtaining a detailed exposure history to assist in the prompt diagnosis of nocardiosis.

## 1. Introduction

Named after the French veterinarian Edmond Isidore Etienne Nocard, who identified a Gram-positive partially acid-fast aerobic bacillus back in 1888, *Nocardia* spp. is the first human aerobic actinomycete described in the literature [1,2]. Until recently, it was considered an opportunistic pathogen as many cases were described in patients with acquired immunodeficiency syndrome (AIDS) and others with impaired cell-mediated immunity (patients with leukemia or lymphoma, transplant recipients, patients on long term glucocorticoid therapy) [3,4,5,6]. Recently, nocardiosis has been reported in patients without underlying co-morbidities and in some studies about one-third of infected patients do not have an identifiable predisposing factor [6]. These seemingly immunocompetent patients might have an underrecognized immunodeficiency [5] but disease course in those patients appeared to be less severe [6].

Nocardiosis is a worldwide disease. The exact incidence of nocardiosis is unknown in United States (U.S.), as it is not a reportable disease. Only 500–1000 cases are diagnosed each year, according to the National Organization for Rare Disorders (NORD). Person-to-person spread is not well documented and nearly all cases are sporadic. The most common sites of infection are the lungs, brain, and the skin. About 80% of nocardiosis presentations are abscesses of the brain, pulmonary abscesses or disseminated infection, while the skin is affected in the remaining 20% of patients [7]. More than 50 species of Nocardia were isolated in the environment, in fresh and saltwater, vegetable matter, and soil with the most common isolates in the U.S. being *N. brasiliensis*, *N. nova*, and *N. farcinica* [6,7,8,9]. Skin involvement in nocardiosis is classified as the following: (1) primary cutaneous, (2) lymphocutaneous, (3) cutaneous manifestations of disseminated nocardia, and (4) mycetoma [7]. Lymphocutaneous nocardiosis (LCN) is defined as the disease where in addition to skin lesions there is an involvement of the regional lymph nodes manifesting as nodular lymphangitis. This syndrome is also called “sporotrichoid nocardiosis” as the clinical pattern is indistinguishable from lymphocutaneous syndrome due to *Sporothrix schenckii* infection [8,9].

## 2. Case Report

A 66-year-old Caucasian male presented to the emergency department (ED) with complaints of worsening pain, swelling, and redness in his left finger and hand. The patient was healthy, did not have any chronic medical problems, and was not taking any medications. He had no history of recurrent infections, did not drink alcohol or use illicit drugs, and was a lifelong nonsmoker.

**Figure 1:**
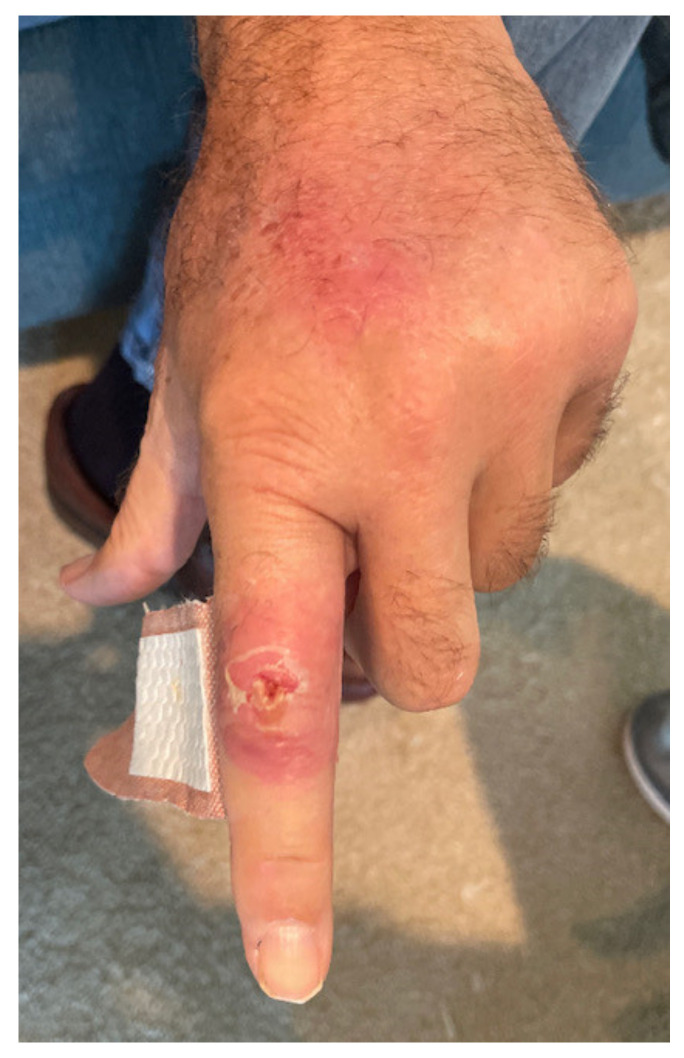
Redness and the swelling of the digit with infection spreading to the dorsum of the hand.

**Figure 2:**
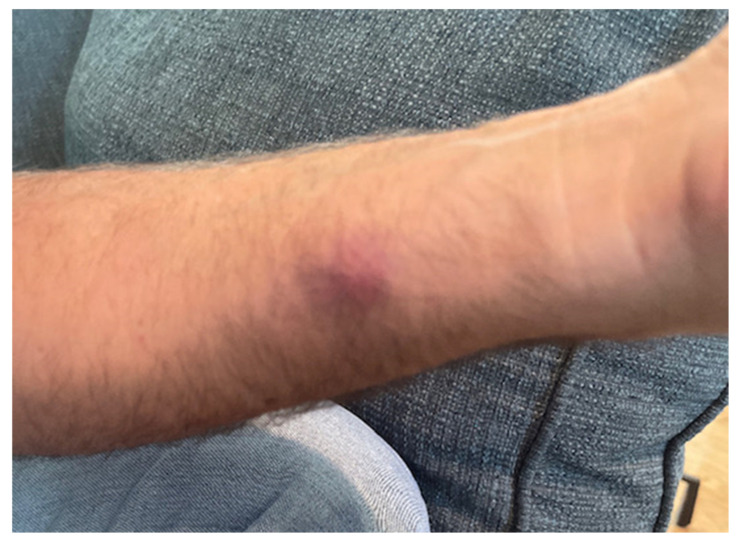
Nodular, lymphatic spread of infection in the forearm.

**Figure 3:**
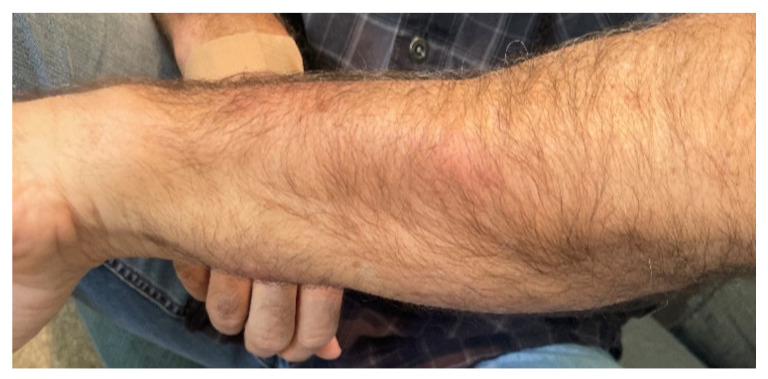
Lymphocutaneous spread manifested as painful, red, and swollen nodular lesion.

**Figure 4:**
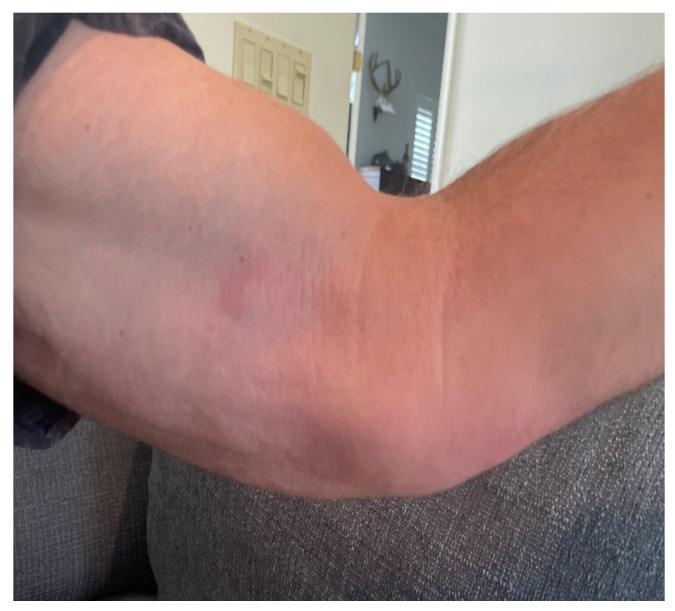
Further spread of infection involving cubital fossa and surrounding lymph nodes.

One week prior to ED presentation, the patient accidentally cut his left index finger with a kitchen knife while working on a pot with garden soil residue. Initially, he was evaluated by his primary care provider and was prescribed trimethoprim-sulfamethoxazole (Bactrim) 1 tablet 160/800 mg twice daily by mouth. Patient took the medication as prescribed for 2 days; however, the swelling and erythema began to spread up the dorsal aspect of the hand and he began noticing swollen lymph nodes in the forearm, elbow, and axilla (Figure 1, Figure 2, Figure 3 and Figure 4). The patient did not have any fevers, chills, or any constitutional symptoms. Because of this clinical progression, he was re-evaluated by his primary care physician and treated with an intramuscular injection of 2 grams of ceftriaxone. He was instructed to stop Bactrim and to start doxycycline 100 mg by mouth two times a day. He took this regimen for 48 h, but due to worsening of the pain and swelling, presented to the ED for further evaluation.

The physical exam in the ED was notable for well-appearing man who was not in any distress. His vital signs were within normal limits. Heart, lung, abdominal, and neurologic exams were also normal. The exam of his left hand and arm was remarkable for an open wound and ulceration on the dorsal aspect of the left hand (approximately 5mm) with erythema extending proximally to the forearm. There was decreased range of motion above the proximal interphalangeal (PIP) joint secondary to tightness and swelling in the finger. There was a slight thick yellow drainage from the wound. At that time, the patient developed chills, but his temperature was normal. Laboratory investigations revealed a white blood cell count of 8.2 × 10^9^/L (reference range: 3.4–9.6 × 10^9^/L), C-reactive protein of 12.6 mg/L (reference range: 0–8 mg/L), normal sedimentation rate of 15 mm/h (reference range: 0–22 mm/h). X-ray of left hand and index finger revealed soft tissue swelling over the dorsal PIP joint, there were no signs of erosive changes, and no other obvious deformity about the hand or left index finger. No foreign bodies were seen. Ultrasound of the left upper extremity was negative for deep venous thrombosis and no drainable fluid collections were noted in the arm. Wound and blood cultures were collected.

**Figure 5:**
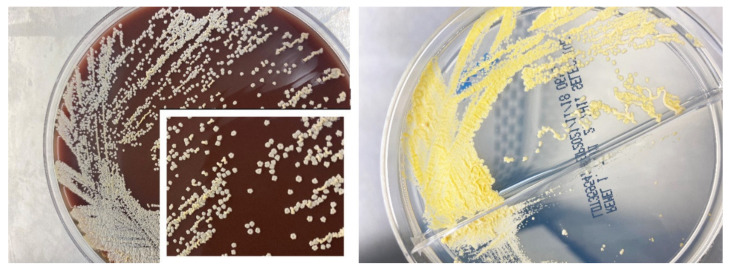
*Nocardia brasiliensis* growing after 5 days of incubation at 35 °C on Chocolate agar. Note the characteristic dry yellow/orange/white colony morphology. Note characteristic dry colonies resembling “molar teeth” (see inset). *Nocardia brasiliensis* growing after 5 days of incubation at 35 °C on Middlebrook 7H11//7H11 Selective Agar. Note the characteristic dry yellow/orange/white colony morphology.

**Figure 6:**
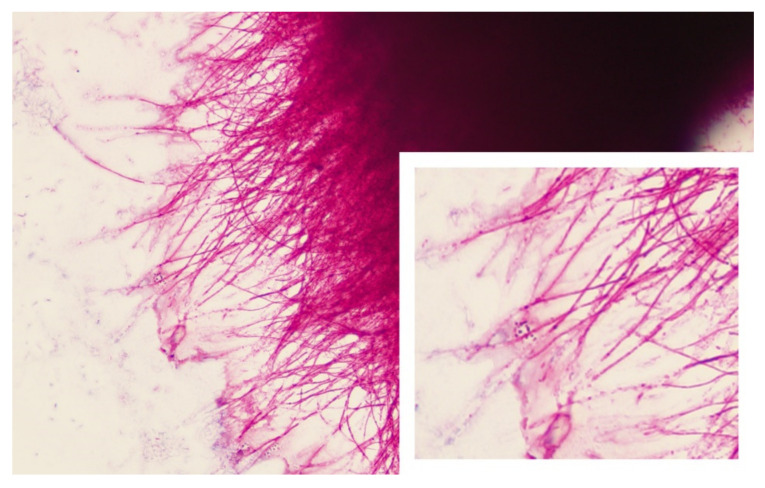
Modified kinyoun stain (×100) of *Nocardia brasiliensis*. Note the branching bacilli and the characteristic cherry red hue of a positive modified kinyoun stain. Bacilli can appear beaded (see inset).

Patient was initiated on clindamycin 600 mg intravenously (IV) every 8 h and ampicillin-sulbactam (Unasyn) 300 mg IV every 6 h for presumable bacterial cellulitis that failed to respond to outpatient treatment. The patient was admitted as an inpatient from the ED to the patient’s home with Mayo Clinic’s home hospitalization program, Advanced Care at Home, and cared for by the hospital internal medicine team. Despite this dual, broad-spectrum antimicrobial therapy, he continued to have progression of the cellulitis, with worsening pain and ongoing chills. Initial blood cultures remained without growth, however due to persistent chills, they were repeated to increase diagnostic yield for possible bacteremia or fungemia. The differential diagnosis was revisited and given the lymphocutaneous pattern of the syndrome, suspicion for *Sporothrix schenckii* infection, mycobacterial infection, and nocardiosis were considered. Around the same time, initial cultures from the left finger grew small white/yellow/orange dry colonies after 48 h of incubation at 35 °C (Figure 5). A Gram stain, modified Kinyoun stain, and a Kinyoun stain were performed to rule out acid-fast or partially acid-fast organisms. The modified Kinyoun stain was identified as positive with a preliminary identification of aerobic actinomycete (Figure 6). Ultimately, the isolate was identified as *Nocardia brasiliensis* by matrix assisted laser desorption ionization-time of flight (MALDI-TOF) mass spectrometry. Therapy with high dose Bactrim (2 tablets orally every 8 h) and ciprofloxacin (500 mg orally two times a day) was started. After 48 h, the pain started to abate, as well as the swelling and redness. Chills resolved and the patient remained afebrile.

Susceptibilities showed resistance to ciprofloxacin which was subsequently replaced with amoxicillin/clavulanic acid (Augmentin) 875/125 mg PO twice daily. The patient completed a total of 8 weeks of therapy and his symptoms completely resolved.

## 3. Discussion

*Nocardia* spp. have an extraordinary capability to escape the immune response but the exact mechanism by which they do so is unclear [10]. Their known virulence factors include cell wall lipids and multiple enzymes, such as catalase, superoxide dismutase, hydrolases, lipase, and proteases [11]. Experiments have shown that even though human-derived alfa defensins and enzymes from neutrophils have killed certain strains of *Nocardia* spp., most strains have survived the interaction with neutrophils and monocytes despite evident phagocytosis of a considerable number of these bacteria [12,13]. Properties which enable *Nocardia* spp. to survive within phagocytes include neutralization of oxidants, prevention of phagosome-lysosome fusion, and/or prevention of phagosome acidification. Hence, neutrophils can ingest Nocardia but in certain cases, they do not kill them [12,13,14]. Therefore, cell-mediated immunity is crucial for definitive elimination of infection. There is an increase in the reports of infection in immunocompetent people, and reasons for this remain unclear. These seemingly immunocompetent individuals (full testing for immunodeficiency is not routinely performed, in the case presented or the ones previously reported) might have some unrecognized primary immunodeficiencies and inborn immunity errors [5,9]. The patient that we report had no underlying medical co-morbidities that would put him at risk for development of infection and was not taking any immunosuppressive medications. HIV infection was ruled out, however, further testing for primary immunodeficiencies in his age group is not a standard practice in U.S. and not performed.

Pulmonary infections are the most common, likely due to the ability of Nocardia to become airborne and avoid the defense of alveolar macrophages. Patients who suffer from chronic lung disease, such as COPD or bronchiectasis, are more likely to become infected given impaired local defense mechanisms, structural modifications of the bronchial architecture, and bacterial colonization of the bronchus which alters ciliary motility and causes epithelial damage [15]. Some experts suggest that the higher incidence of infection in patients with COPD might be due to frequent use of steroids in addition to structural changes of lung parenchyma [9].

Skin infections usually occur following a traumatic inoculation during an insect bite, animal scratch, puncture wound, or gardening. Men have a three times higher likelihood of being infected than women, with middle-aged men working outdoors having the highest risk [16]. Some variations between the *Nocardia* spp. exist. For example, *N. asteroides* tends to disseminate more than *N. brasiliensis*, while *N. brasiliensis* is more commonly implicated as the pathogen in cutaneous infections [6,9,17,18]. Our patient had a definite exposure while gardening, including a penetration injury by a soil contaminated knife. Unfortunately, this exposure history went unrecognized in initial visits, which contributed to the delay in diagnosis. His demographics (age and gender) and exposure to garden soil, in retrospect, should have alerted clinicians that the infection may be nocardiosis. Blood cultures remained negative, which is not surprising as Nocardia bacteremia is less common [9,17].

We have searched the PubMed database for case reports and case series of *N. brasiliensis* lymphocutaneous infection using the key words *Nocardia brasiliensis* and skin or lymphocutaneous. Our search yielded 17 cases [19,20,21,22,23,24,25,26,27,28,29,30] that are summarized in Table 1. Of these cases, most patients were men (90%), with only two infections reported in women. Immunosuppression was present in six patients (32%) while the others were immunocompetent. Most of the patients were treated with Bactrim, and duration of treatment was from one month to six months in patients who were not immunocompromised. In immunosuppressed patients, treatment was longer, ranging from six months to a year, and in one case the treatment was indefinite due to ongoing immunosuppression.

Diagnosis of nocardiosis is based on demonstrating the organism in the clinical specimen. On the Gram stain or modified Kinyoun stain (less sensitive), *Nocardia* spp. appears as a Gram-positive, filamentous rod with hyphae like branching [9,17]. Molecular biology techniques are the gold standard for the diagnosis. Nocardia PCR based assays targeting the rrs gene and/or MALDI TOF mass spectrometry are used to identify Nocardia species [17].

Dermal features and their associated histopathologic changes may be seen with other infections, so maintaining a broad differential becomes key when evaluating these lesions. Grossly, lymphocutaneous nocardiosis most strongly resembles Sporotrichosis, *Erysipelothrix rhusiopathiae*, and, to an extent, *Francisella tularensis* infection in both the lymphangitic spread and propensity to involve otherwise healthy individuals [31]. Similarly, the presence of a mixed inflammatory infiltrate with neutrophilic aggregate suggestive of micro-abscesses is conserved, the exception being the propensity of caseating granuloma in lymphangitic tularemia [32,33]. Nodular, sporotrichoid, verrucous lesions reminiscent of lymphocutaneous nocardiosis are seen in cutaneous leishmaniasis with conserved features of mixed inflammatory infiltrate; even the later stages of granulomatous disease may yet resemble actinomycetoma [34]. Cutaneous mold infections (i.e., Aspergillus, *Scedosporium* spp., etc.) are grossly and histopathologically similar to actinomycosis, particularly given the propensity towards chronic granulomatous disease (mycetoma formation) [35]. Non-tuberculous mycobacterial lymphocutaneous spread, most notably *M. marinum*, can be associated with similar, suppurative findings on histopathology, particularly in the early phase before organization into tuberculoid granulomas [36].

Additionally, mycetoma, which is a chronic, primary skin infection located in the dermis and subcutaneous tissue and affects usually immunocompetent patients in tropical and sub-tropical countries should be kept in mind [37]. A meta-analysis of 8763 cases of mycetoma, reported since 1944, found *Nocardia brasiliensis* responsible for 5.76% of the cases [38]. In our patient, the lesions developed acutely so mycetoma was ruled out based on the timeline and duration of the infection.

The fact that our patient was correctly diagnosed only when he was evaluated for the fourth time illustrates how difficult it may be for clinicians to accurately diagnose these infections. It further emphasizes the importance of obtaining an exposure history as well as a tissue biopsy for more definitive diagnosis with suspect lesions that are unresponsive to initial, empiric therapy [38] Particularly noteworthy is that this patient was correctly diagnosed while admitted to a home hospitalization program in which the history and physical exam was performed through a hybrid model of in-person and virtual care.

Isolation of Nocardia from a clinical specimen should not be regarded as a contaminant (particularly in immunocompromised patients) and must be treated in all cases [9]. There is not an established treatment regimen for Nocardiosis given variable susceptibility patterns [39] although sulfonamides are often used. Hence, it is important to follow antimicrobial susceptibility results to treatment option to patients. In the U.S., the most commonly used sulfonamide preparation is Bactrim, which is active against most Nocardia species [17,18,40]. Alternative antibiotics include amikacin, ceftriaxone, cefotaxime, minocycline, moxifloxacin, linezolid, tigecycline, amoxicillin-clavulanic acid, and carbapenems of which imipenem is the most active of its group [9,18]. Linezolid is considered an excellent alternative or as an addition to Bactrim for initial empiric treatment, although myelosuppression and hepatotoxicity are common side effects, particularly with prolonged use.

The initial empiric antimicrobial regimen in our patient was Bactrim. Although appropriate, it failed most likely due to inadequate dosing. He was treated with one double-stranded tablet two times a day, which is infective for the treatment of nocardiosis. Once switched to two double stranded tablets every 8 h, he showed remarkable clinical improvement.

Most of the cases are treated empirically with a combination drug regimen which provides enhanced activity against Nocardia and increases the chances that at least one of the medications in the regimen will be effective. In an immunocompetent patient with cutaneous infection, monotherapy with Bactrim or a combination regimen may be effective and should continue for 2–6 months. Some cases, depending on the extension and complications, such as mycetoma and deep abscesses, may require surgical treatment. A three-drug regimen) including Bactrim, amikacin, and either ceftriaxone or imipenem has been used in cases with central nervous system (CNS) involvement, disseminated infection, and complicated disease. In such cases, longer duration of therapy up to a year may be necessary [9,17]. Prolonged treatment duration is used to avoid relapses and should be based on patient’s immune condition and extent of disease [7,13,40,41].

## 4. Conclusions

To conclude, lymphocutaneous nocardiosis is rare, and most commonly occurs in immunocompetent men. The differential diagnosis is complex and a high index of suspicion is needed to diagnose this infection. Appropriate exposure history is crucial for diagnosis.

## Figures and Tables

**Table 1:** Summarize all published cases of lymphocutaneous *Nocardia brasiliensis* infection.

Case/Ref	Age	Sex	Country	Comorbidities	Immuno-suppression	*Nocardia* spp.	Type of Infection	Treatment (Duration)	Outcome
1 [19]	64	M	Greece	None	No	NB	LC	2 months	Successful
2 [20]	49	M	USA	DM	No	NB	LC	6 months	Successful
3 [20]	59	M	USA	HTN, Metastatic Esophageal Cancer	Yes	NB	LC	6 months	Successful
4 [20]	78	M	USA	CAD, Prostate Cancer	Yes	NB	LC	6 months	Successful
5 [20]	62	M	USA	Ulcerative colitis	Yes	NB	LC	Indefinite Bactrim due to immuno-suppressive regimen	Successful
6 [21]	32	M	Unknown	None	No	NB	LC	3 months	Successful
7 [22]	34	M	Chinese	None	No	NB	LC	6 weeks	Successful
8 [23]	65	M	Unknown	PKD s/p renal transplant	Yes	NB	LC	1 year	Successful
9 [24]	65	M	Unknown	Myasthenia gravis	Yes	NB	LC	6 months	Successful
10 [25]	37	M	USA	None	None	NB	LC	2 months	Successful
11 [25]	69	F	USA	DM, Temporal arteritis	Yes	NB	LC	3 months	Successful
12 [25]	70	M	USA	CAD, PVD, HTN	No	NB	LC	2 months	Successful
13 [26]	61	M	Brazil	None reported	No	NB	LC	14 days, pt refused longer treatment.	Successful
14 [27]	45	M	USA	None reported	No	NB	LC	3 months	Successful
15 [28]	82	F	Israel	HTN, HLD, CVA, Osteoporosis	No	NB	LC	Bactrim discontinued due to allergic reaction. Doxycycline prescribed, unknown duration.	Successful
16 [29]	9	M	Australia	None	No	NB	LC	6 months due to relapsing of the infection	Successful
17 [30]	87	M	China	CAD, asthma	No	NB	LC	4 weeks	Successful

DM—diabetes mellitus; HTN—hypertension; M—male; F—female; CAD—coronary artery disease; PKD—polycystic kidney disease; NB—*Nocardia brasiliensis*, LC—Lymphocutaneous; PVD—Peripheral vascular disease; HLD—hyperlipidemia; CVA—cerebrovascular accident; TMP/SMX—Trimethoprim-Sulfamethoxazole; IS—immunosuppression; USA—United States of America.
